# High rate of unintended pregnancy among pregnant women in a maternity hospital in Córdoba, Argentina: a pilot study

**DOI:** 10.1186/1742-4755-6-11

**Published:** 2009-07-20

**Authors:** Celina Palena, M Valeria Bahamondes, Verónica Schenk, Luis Bahamondes, Julio Fernandez-Funes

**Affiliations:** 1 Maternity and Neonatal Hospital, Córdoba, Argentina; 2 Department of Obstetrics and Gynecology, School of Medical Sciences, University of Campinas (UNICAMP), Campinas, Brazil

## Abstract

**Background:**

Although Argentina has a new law on Reproductive Health, many barriers continue to exist regarding provision of contraceptive methods at public healthcare facilities.

**Methods:**

We asked 212 pregnant women selected at random at the Maternity and Neonatal Hospital, Córdoba, Argentina, to participate in our descriptive study. Women were asked to complete a structured questionnaire. The objectives were to determine the rate of unintended pregnancies, reasons for not using contraception, past history of contraceptive use, and intended future use.

**Results:**

Two hundred women responded to the questionnaire. Forty percent of the women stated that they had never used contraception and pregnancy was declared unintended by 65%. In the unintended pregnancy group, almost 50% of women said that they had not been using a contraceptive method because they were "unaware about contraception", and 25% stated that their contraceptive method had failed. Almost 85% of women stated that they intended to use contraception after delivery.

**Conclusion:**

Approximately two-thirds of all pregnancies in this sample were unintended. Although the data is limited by the small sample size, our findings suggest that our government needs to invest in counseling and in improving the availability and access to contraceptive methods.

## Background

Governmental support for family planning (FP) activities is relatively new in Argentina, since for many years neither the federal, state nor municipal governments provided contraceptive methods in their healthcare facilities. An extreme example was the province of Córdoba, which is ranked second highest for its economic performance in the country, where, in the middle of the 60's, the use of any modern contraceptive method was prohibited by a law passed during the military dictatorship. Even after the democracy was reestablished, conservative members of society succeeded in upholding this restrictive policy. In May 2003 the federal government obtained congressional approval of a reproductive health law that included the provision of modern contraceptive methods at public health facilities [1]. Almost all the provinces followed federal guidelines and implemented local legislation.

Nevertheless, many barriers continue to exist for the provision of contraceptive methods at public healthcare facilities. Although contraceptive methods are currently provided at many of these facilities, little has been done with respect to training of healthcare providers in counseling, other activities related to FP and the logistics related to the use of different contraceptive methods. Consequently, contraceptive methods may be used incorrectly or inconsistently [2], mainly methods such as oral and injectable contraceptives. These methods are more demanding on a woman's day-to-day participation and may therefore increase the failure rate.

The objectives of this exploratory and descriptive study were to evaluate a group of pregnant women to determine in how many cases the current pregnancy was unintended, to list the reasons reported by these women for not using contraception, to evaluate previous contraceptive use, and the intention of contraceptive use after the current pregnancy. The findings of this study could be useful to policy makers in improving FP programs.

## Methods

The study was conducted at the Maternity and Neonatal Hospital of the Province of Córdoba, Argentina and the protocol was approved by the Institutional Review Board. A total of 212 women were invited to participate in the pilot study that took place between December 2006 and June 2007.

Women were contacted at random at the prenatal clinic and among women hospitalized regardless of gestational age. Twelve women refused to participate, consequently, 200 women (94%) were included. Participants were interviewed face-to-face by two of the authors using a pre-tested questionnaire developed by the authors. The questionnaire covered socio-demographic information (age, education, working status), reproductive history, past contraceptive practice during lifetime, alcohol and smoking habits, and history of sexual and physical violence. Questions focused on whether or not the woman had intended to become pregnant at the time of conception, and whether she intended using contraception following delivery. Women who stated that their current pregnancy had been unintended were asked to provide the reasons for not having used contraception at the time of conception.

Assessing whether or not a pregnancy is 'unintended' is a difficult task and involves well-known methodological problems [3-6]. We therefore constructed an "unintended pregnancy, yes/no" variable for which the woman's answer to the following question was used: "Was it your intention to become pregnant (at that time)? Earlier/at that time/later/not at all/you have never thought about it" [7].

The statistical analysis includes a description of the socio-demographic characteristics of the participants. The reasons for not using contraception and the factors contributing to contraceptive failure were analyzed for all women who stated that the current pregnancy was unintended.

## Results

Of the 200 women interviewed, 130 stated that their current pregnancy had been unintended, while the remaining 70 reported that they had intended to get pregnant. The mean age was 25.9 (SD ± 7.0 years; range 14–42) and 25.7 (SD ± 7.0 years; range 16 – 40) among women in the intended and unintended pregnancy group, respectively. The two groups were similar with regard to educational level, occupation, smoking status and history of sexual and/or physical violence. A higher proportion (83.4%) of women whose pregnancy had been intended reported living with a partner (Table 1).

**Table 1:** Selected socio-demographic characteristics of the sample population according to whether or not the pregnancy was intended.

Variables	Intended (n = 70)	Unintended (n = 130)	*P*-value
Age (years ± SD)	25.9 ± 7.0	25.7 ± 7.0	0.8597*
*Education n (%)*			0.2532***
Some elementary school education	10 (14.3)	8 (6.2)	
Completed elementary school	15 (21.4)	24 (18.5)	
Some high school education	26 (37.1)	65 (50.0)	
Completed high school	14 (20.0)	23 (17.7)	
Some college education	5 (7.1)	10 (7.7)	
*Marital status n (%)*			0.0040***
Without a partner	13 (18.6)	53 (40.8)	
Living with a partner	57 (83.4)	77 (59.2)	
*Occupation n (%)*			1.0000**
Housewife	54 (77.1)	97 (74.7)	
Employed outside the home or student	16 (22.9)	32 (23.9)	

* Mann Whitney non-paired test; **Fisher's Exact test; ***Chi-square test

Women whose pregnancy was unintended had a statistically significantly higher number of previous pregnancies and deliveries and had more live children compared to women with intended pregnancies. Moreover, age at sexual debut was lower in the unintended pregnancy group (Table 2).

**Table 2:** Some variables regarding pregnancy and sexual history according to whether or not the current pregnancy was intended.

Variables	Intended* (n = 70)	Range	Unintended* (n = 130)	Range	*P*-value**
Gravida	1.6 ± 0.2	1 – 7	2.6 ± 0.2	1 – 11	0.0115
Para	1.2 ± 0.2	1 – 5	2.1 ± 0.2	1 – 9	0.0095
Abortion	0.3 ± 0.1	0 – 2	0.4 ± 0.1	0 – 5	0.5211
Live children	1.1 ± 0.2	0 – 5	2.1 ± 0.2	0 – 9	0.0035
Age at sexual debut	17.0 ± 0.4	12 – 30	15.9 ± 0.2	11 – 25	0.0089
Number of lifetime partners	2.5 ± 0.2	1 – 7	2.6 ± 0.2	1 – 20	0.6031

* Values are mean ± SEM. ** Mann Whitney non-paired test

Twenty-nine (41.4%) of the women in the intended pregnancy group and 50 (38.5%) in the unintended pregnancy group declared they had never used contraception before. The mean age of these women was similar between the groups (23.7 (SD ± 5.5; range 16 to 40) for the intended and 23.9 (SD ± 7.8; range 15 to 42 y for the unintended pregnancy group). In case of contraceptive use, the most common methods were combined oral contraceptives (COC) and condoms. When asked about their intention for future contraceptive use, the two methods most often mentioned were COC and intrauterine devices (IUD) (Table 3). Almost 50% of women in the unintended pregnancy group, who did not use contraception, stated that they were "unaware about contraception" (Table 4). One in every four women whose pregnancy was unintended stated that their contraceptive method had failed.

**Table 3:** Lifetime history of contraceptive use and intention to use contraceptives after the present pregnancy according to whether or not the current pregnancy was intended.

	*Intended* *(n = 70)*	*Unintended* *(n = 130)*
*Lifetime method used before this pregnancy*	N (%)	N (%)

Oral contraceptive	20 (28.6)	25 (19.2)
Condom	8 (11.4)	30 (23.1)
IUD^#^	5 (7.1)	9 (6.9)
Injectable	6 (8.6)	5 (3.8)
Rhythm method	2 (2.9)	11 (8.5)
Never user	29 (41.4)	50 (38.5)
		
*Intention to use*		
Oral contraceptive	23 (32.9)	31 (23.8)
IUD^#^	22 (31.4)	49 (37.7)
Sterilization	5 (7.1)	24 (18.5)
Injectable	6 (8.6)	6 (4.6)
Condom	3 (4.3)	2 (1.5)
Do not know	11 (15.7)	18 (13.8)

^#^IUD: Intrauterine device

**Table 4:** Reasons for not using a contraceptive method given by the women whose pregnancy were unintended.

*Reasons*	*N (%)*
Unaware about contraception	59 (45.3)
Contraceptive failure	33 (25.4)
Inappropriate use of contraceptive method	19 (6.9)
Waiting for a method*	8 (6.1)
"Taking a break" from contraceptive use	5 (3.8)
Afraid of side effects with contraception	4 (3.1)
Unable to attend the healthcare facility	2 (1.5)

* Waiting for IUD insertion or tubal ligation

## Discussion

This study was conducted in the largest maternity hospital in the province of Córdoba, Argentina were almost 7,000 deliveries are performed annually. The most important finding was the fact that 65% of respondents stated that their current pregnancy was unintended. Overall, according to their answers, these women had more years of schooling and fewer of these women had a stable partner at the time of the interview compared to those whose pregnancy was intended.

The limitations of this study were mainly due to the small sample size and the convenience sample which may not be representative of the general situation at the province or at the country. Argentina has a population of almost 40 million and a total fertility rate (TFR) of 2.5. A woman's lifetime risk of dying from maternal causes is 1 in 530. Contraceptive prevalence among women of 15–49 years of age is 65.3% as reported by the government. According to the 2001 statistics, the distribution of contraceptive use was 30.4% for COC, 22% for condoms, 9.5% for IUDs, and 13.5% for traditional methods [8]. Despite this high contraceptive prevalence and relatively low TFR, the results of our exploratory study are not consistent with these figures. The data collected may not represent the actual situation of these women, since some may have been embarrassed to say whether their pregnancy was intended or unintended [3]. This situation could reflect on the low number of abortions reported by the women.

Nevertheless, the fact that almost two-thirds of women reported that their pregnancy was unintended is an information that should alert health authorities and mobilize actions to improve contraceptive services. Data from France showed that between 20 and 33% of the annual births are unplanned or unintended [7,9], and in Edinburgh, Scotland this figure is only 28% [10,11]. In the United States, despite the high contraceptive prevalence the unintended pregnancy rate is still high with almost half (49%) of all pregnancies reported to be unintended [12]. The disparity between European countries and the United States could be explained because in European countries women have a broader choice of contraceptive methods and many of them were reimbursed by the social security; however, in the United States, the range of contraceptives is restricted and in many cases the cost is inaccessible to low-income women [13].

In our sample, among the women whose pregnancy was unintended, the main reasons for not having used contraception were: "that they were unaware about contraception", "contraceptive failure", "inappropriate use", and "waiting to be provided with a method". Similar results were observed in a study from France [7]. Contraceptive failure could occur with all contraceptive methods although is rare with long-acting methods and "perfect use" methods such as the IUD and subdermal implants [14]. However, among the women in our sample who confirmed the use of contraception in the past, the two most common methods used were COC and condoms, both of which have a higher probability of method failure [15]. A study conducted in Norway with women who claimed to have become pregnant during COC use revealed that some of these women had stopped using the pill prior to their last menstrual period, while others were unable to supply any information at all on their use of COCs or to provide the prescription for COC use given just prior to pregnancy [16].

It should also be taken into account that many cases of unintended pregnancy could result in abortion. The fact that abortion is illegal in Argentina and access to safe abortion services is severely restricted, especially for women from low socioeconomic background may have contributed to the high number of unintended pregnancies in our sample. Our results show that of women who declared not having used contraception, 43.8% stated as their main reason the fact that they "were unaware about contraception". This reason could be interpreted in a similar way as "*did not believe I could become pregnant then*" as the main reason for not having used contraception observed in a Swedish study with women who requested abortion (and could be interpreted as unintended pregnancy) [17]. We believe that both reasons given by women could be interpreted in a similar way, "unaware about contraception".

The issue of unintended pregnancy is a challenge in many countries. The first step towards providing contraceptive methods is to implement an appropriate legal and political framework, which was established in Argentina by creating the law on reproductive health. However, this is only the first step. Contraceptive prevalence depends on other issues. One of them is the provision of methods at low or no cost to low-income women such as the women in the present study. In the US, a link has been found between unplanned pregnancies and abortions and low-income women [18], and legislation has been approved to help reduce the gap between women of different socioeconomic levels [19].

Women with no healthcare insurance, attend public health sector facilities where FP services are mainly provided by medical doctors and at hospitals. These women need to schedule an appointment for a consultation, generally this leads to long delays during which women are unprotected and may become pregnant. Another situation to take into account is that often the logistics of contraceptive supply are ineffective, and in many clinics certain contraceptive methods may be unavailable. Although legislation states that the provision of contraceptives is free of charge at public healthcare facilities, in many cases this policy is not effectively implemented and underprivileged women may still be required to pay for contraceptive services despite the supposedly universal access to contraception [20].

Service providers contribute towards the difficulties attempting to obtain contraception because they may not be sufficiently trained to provide enough information needed to choose a contraceptive method and to use it correctly. In our study, some women, of the group of unintended pregnancy, referred as one of the reasons for not using any contraceptive method was that they were "waiting for a method" and probably they were referring to IUD insertion or tubal ligation [21]. Poor women seeking public healthcare are often not in a position to challenge medical authority or question doctors' decisions, and physicians need to make an effort to listen to women and allow them to express their needs [22]. Contraceptive failure has been reported to be lower when medical doctors take time to talk to their patients [23].

Family planning services not only depend on the availability of contraceptive methods, services, and training of the service providers, it is also important to offer information to the general population and especially in schools. The development and distribution of information, education and communication materials represent another challenge for governments and health policy makers. Lack of information may increase the rate of contraceptive failure. In the current sample, more than 40% of women whose pregnancies were unintended reported that they had not been using a contraceptive method because they were unaware that they needed contraception.

The majority of women seeking contraceptive services are healthy and their objective is to avoid a pregnancy. In the countries in which abortion is legal, women have a broader choice; however, in countries such as Argentina where abortion on request is unavailable, women have to continue with the pregnancy or undergo abortion which may be under unsafe conditions with the risk of severe complications.

## Conclusion

Our findings show that almost two-thirds of the women interviewed in the largest maternity hospital in Cordoba, Argentina stated that their current pregnancy was unintended and it is not in agreement with currently available data on contraceptive prevalence. The main reason for unmet need at the time of conception was that women were unaware about contraception. Although our data were limited by the small sample size, it provides information to authorities and reinforces the need to invest in counseling on contraceptive methods in order to reduce incorrect method use, and make contraceptive methods available, and accessible to reduce the unmet needs.

## Abbreviations

COC: combined oral contraceptives; IUD: Intrauterine devices; TFR: Total fertility rate

## Competing interests

The authors declare that they have no competing interests.

## Authors' contributions

All the authors contributed equally in the design, interpretation of the data and review and revise the final manuscript.
